# CuS nanoparticles and camptothecin co-loaded thermosensitive injectable hydrogel with self-supplied H_2_O_2_ for enhanced chemodynamic therapy

**DOI:** 10.3389/fbioe.2022.1003777

**Published:** 2022-08-29

**Authors:** Wenxue Tang, Xiang Li, Zeming Liu, Lyu Meng, Daoming Zhu, Qinqin Huang

**Affiliations:** ^1^ The Research and Application Center of Precision Medicine, The Second Affiliated Hospital, Zhengzhou University, Zhengzhou, China; ^2^ Department of Central Laboratory and Precision Medicine Center, Department of Nephrology, The Affiliated Huai’an Hospital of Xuzhou Medical University and Huai’an Second People’s Hospital, Xuzhou, China; ^3^ Department of Plastic Surgery, Tongji Hospital, Tongji Medical College, Huazhong University of Science and Technology, Wuhan, China; ^4^ Department of Radiation and Medical Oncology, Hubei Key Laboratory of Tumor Biological Behaviors, Hubei Cancer Clinical Study Center, Zhongnan Hospital of Wuhan University, Wuhan, China; ^5^ Department of General Surgery and Guangdong Provincial Key Laboratory of Precision Medicine for Gastrointestinal Tumor, Nanfang Hospital, The First School of Clinical Medicine, Southern Medical University, Guangzhou, Guangdong, China

**Keywords:** chemodynamic therapy, hydrogel, CuS NPs, camptothecin, self-supplied H_2_O_2_

## Abstract

Chemodynamic therapy (CDT) is a kind of anti-tumor strategy emerging in recent years, but the concentration of hydrogen peroxide (H_2_O_2_) in the tumor microenvironment is insufficient, and it is difficult for a single CDT to completely inhibit tumor growth. Here, we designed a CuS nanoparticles (NPs) and camptothecin (CPT) co-loaded thermosensitive injectable hydrogel (SCH) with self-supplied H_2_O_2_ for enhanced CDT. SCH is composed of CuS NPs and CPT loaded into agarose hydrogel according to a certain ratio. We injected SCH into the tumor tissue of mice, and under the irradiation of near-infrared region (NIR) laser at 808 nm, CuS NPs converted the NIR laser into heat to realize photothermal therapy (PTT), and at the same time, the agarose hydrogel was changed into a sol state and CPT was released. CPT activates nicotinamide adenine dinucleotide phosphate oxidase, increases the level of H_2_O_2_ inside the tumor, and realizes the self-supply of H_2_O_2_. At the same time, CuS can accelerate the release of Cu^2+^ in an acidic environment and light, combined with H_2_O_2_ generated by CPT for CDT treatment, and consume glutathione in tumor and generate hydroxyl radical, thus inducing tumor cell apoptosis. The SCH system we constructed achieved an extremely high tumor inhibition rate *in vitro* and *in vivo*, presenting a new idea for designing future chemical kinetic systems.

## Introduction

By regulating the level of reactive oxygen species (ROS) in tumor cells, changing the redox balance in cancer cells, thereby inducing oxidative damage and death of cancer cells, is one of the effective methods for tumor therapy (Zhang et al., 2020; Zhu et al., 2020; Chen et al., 2021a; Wang et al., 2021a; Zhu et al., 2021a; Zhu et al., 2021b; Zhu et al., 2022a). Chemodynamic therapy (CDT) is a kind of anti-tumor strategy emerging in recent years (Lin et al., 2020; Sang et al., 2020; Chen et al., 2021b; Wang et al., 2021b; Chen et al., 2021c; Zhao et al., 2022). Generally, nano-catalyzed systems are used to induce *in situ* Fenton or Fenton-like reactions (In 1894, HJ·HFenton first pointed out that hydrogen peroxide has a strong oxidizing ability under the catalysis of Fe^2+^, which can oxidize a variety of organic substances (Fenton, 1894); Fenton reagent is the combination of hydrogen peroxide (H_2_O_2_) and Fe^2+^, in which Fe^2+^ is mainly used as a homogeneous catalyst, while H_2_O_2_ plays an oxidizing role. At the same time, in the study of Fenton method, it is found that in addition to Fe^2+^ can catalyze the decomposition of H_2_O_2_ to produce hydroxyl radical (•OH), other transition metal ions such as Mn^2+^, Cu^2+^, Co^2+^, etc. Can also accelerate or replace Fe^2+^ to play this role. Catalysis to achieve oxidation.) in tumors, and the weakly oxidizing H_2_O_2_ is catalyzed (Li et al., 2021a; Deng et al., 2022). The process of transforming into strong oxidative active species such as •OH, •OH can not only damage the DNA chain, but also activate caspase-3 that promotes apoptosis, leading to programmed cell death of cancer cells (Zhang et al., 2019; Zhu et al., 2022b). Notably, this process requires no external stimulus. In contrast to other therapeutic strategies, CDT that is driven by endogenous chemical energy in the tumor microenvironment can successfully prevent oxidative damage to healthy tissues and is therefore tumor-specific (Dong et al., 2018; Liu et al., 2021a). Further, CDT inhibits energy loss during therapy since it does not require sufficient oxygen (O_2_) or external energy input (Feng et al., 2020). However, the insufficient concentration of H_2_O_2_ in the tumor microenvironment severely limits the therapeutic effect of chemokinetic (Zhou et al., 2021; Zhu et al., 2022c). At the same time, CDT alone does not effectively trigger immunological responses (Zhu et al., 2022b), therefore, it is very necessary to achieve high expression of H_2_O_2_ in the tumor microenvironment through a safe and effective method, and to promote the therapeutic effect of CDT through combined therapy, such as radiotherapy and photothermal therapy.

Camptothecin (CPT) is a class of alkaloids isolated from the traditional Chinese medicine Camptotheca. It has natural anti-tumor activity (Tang et al., 2019). Its main anti-tumor mechanism is to inhibit DNA synthesis by inhibiting the activity of S-phase enzyme I in the DNA replication of tumor cells, thereby mediating tumor cell apoptosis. Currently, CPT analogs (ie, irinotecan and topotecan) have been approved by the FDA for cancer treatment. However, CPT has poor water solubility and strong drug resistance, so it has certain limitations in clinical use. But surprisingly, CPT can also increase H_2_O_2_ levels of tumor cells via activation of nicotinamide adenine dinucleotide phosphate oxidase (Zhu et al., 2022b). By co-encapsulating Pd-C SAzymes and CPT in agarose hydrogel, Zhu et al. developed a light-controlled oxidative stress amplifier system that demonstrates improved synergistic anticancer action by creating H_2_O_2_ on its own and trans-forming “cold” tumors (Zhu et al., 2022b). The Pd-C SAzyme in this nanozyme hydrogel system transforms near-infrared laser into heat, which leads to agarose breakdown and subsequent CPT release. Through the activation of nicotinamide adenine dinucleotide phosphate oxidase, the CPT raises the amount of H_2_O_2_ in tumors while enhancing the catalytic activity of SAzymes with peroxidase-like action. In addition, combining photothermal therapy, chemotherapy and nanozyme-based catalytic therapy further promotes the immunogenic death of tumors and improves antitumor immunity. These outcomes demonstrate the synergistic antitumor capabilities of the new SAzyme/chemotherapeutics-based hydrogel system. Therefore, CPT may synergize with CDT to achieve self-supplied H_2_O_2_, effectively solving the problem of poor therapeutic effect of CDT due to insufficient concentration of H_2_O_2_ in the tumor microenvironment.

Semiconductor copper sulfide nanoparticles, with their superior electrical, optical and catalytic properties, have attracted extensive interest in the fields of photodegradation of pollutants, biomarkers, laser monitoring, and DNA detection (Liu et al., 2021b; Ding et al., 2022). As a semiconductor crystal material, CuS nanoparticles (NPs) have strong near-infrared absorption, and the main mechanism is the transition between electron d-d energy levels (Liu et al., 2015). Therefore, the absorption peak of CuS nanoparticles does not vary with the size of the particles. And shape, etc., have good photothermal stability. At the same time, CuS NPs has a strong local surface plasmon resonance effect in near-infrared (NIR), and also has a strong photothermal conversion efficiency, which also provides conditions for its application in photothermal therapy (PTT) (Wei et al., 2018). PTT is a non-invasive treatment (Li et al., 2017a; Li et al., 2017b; Li et al., 2021b; Li et al., 2021c; Li et al., 2022a; Li et al., 2022b). Li et al. prepared thioglycolic acid-modified CuS NPs by wet chemical method, and applied CuS NPs to photothermal therapy of tumors for the first time (Li et al., 2010). The particle size of the CuS NPs is about 3 nm, and it has a strong absorption peak in the NIR, with the maximum absorption peak at about 900 nm. Further MTT experiments show that this CuS NPs has outstanding photothermal treatment effect and good biocompatibility, they pointed out This CuS NPs preparation process is simple, low cost, has a small particle size, and has the advantages of unique optical properties and low biotoxicity. In addition, the Fenton-like reaction between Cu^2+^ ions released in the slightly acidic environment of the tumor and the endogenous H_2_O_2_ of the tumor leads to the generation of hydroxyl radicals for chemokinetic therapy (Ding et al., 2022; Meng et al., 2022), thereby inducing apoptosis. Studies have shown that ROS generated through the Fenton reaction may sensitize tumor cells to chemotherapeutic drugs and promote apoptosis of drug-resistant cells by destroying cytokines or nucleic acids (Cheng et al., 2021; Fu et al., 2021; Yan et al., 2022). Cu^2+^ can also oxidize GSH to oxidized glutathione (GSSG), which further promotes the generation of •OH (Meng et al., 2022). Therefore, CuS NPs can overcome the problem that single chemokinetic therapy is difficult to completely inhibit tumor growth.

**SCHEME 1:**
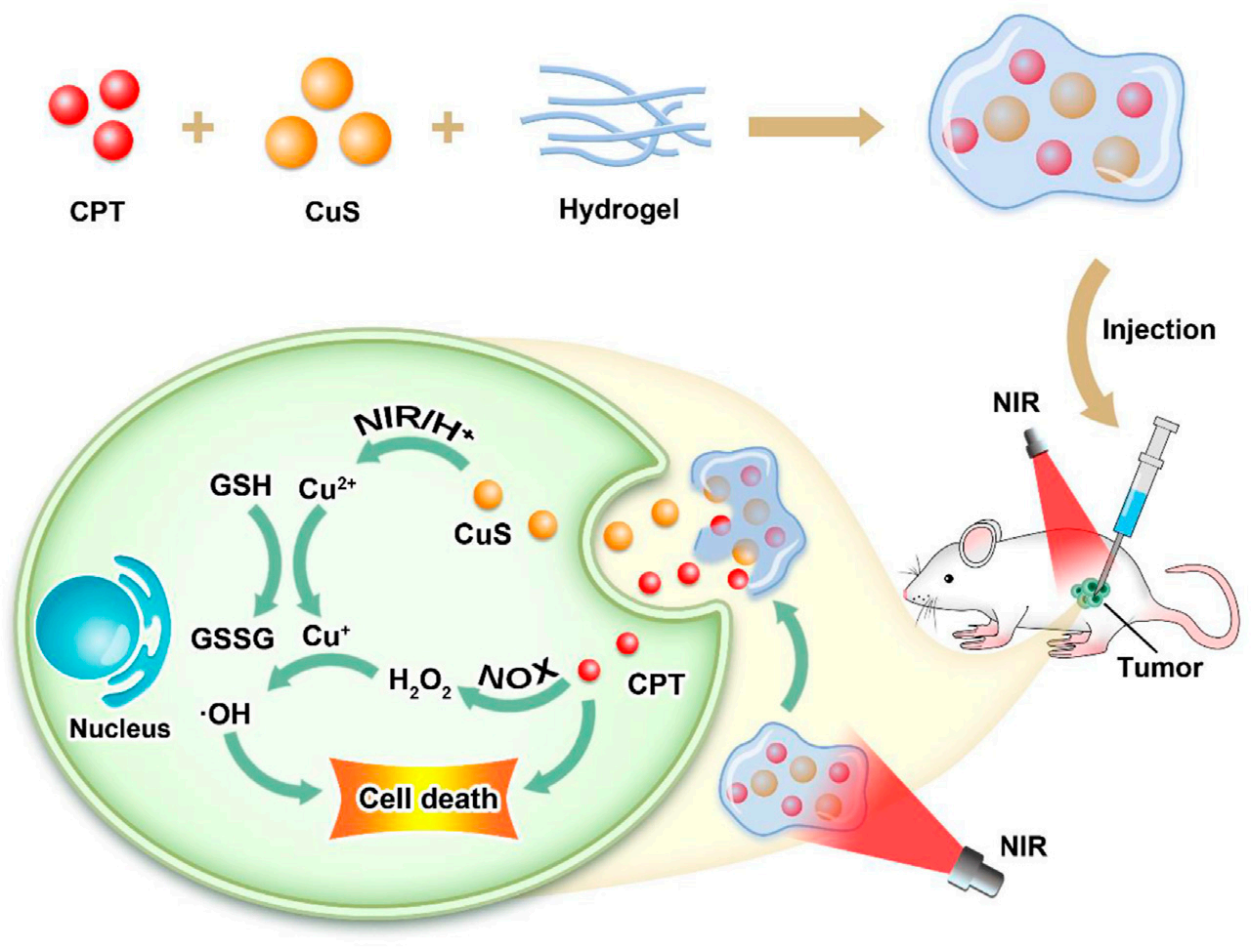
Schematic illustration of CuS NPs and CPT co-loaded thermosensitive injectable hydrogel with self-supplied H_2_O_2_ for enhanced CDT.

Herein, we designed a CuS NPs and CPT co-loaded thermosensitive injectable hydrogel (SCH) with self-supplied H_2_O_2_ for enhanced CDT (Scheme 1). SCH is composed of CuS NPs and CPT loaded into agarose hydrogel according to a certain ratio. The hydrogel gradually solidifies after being injected into the tissue and is able to reside in the body for a long time. Therefore, it can be used repeatedly after one injection. The drug release rate can also be changed by adjusting the laser intensity, laser irradiation cycle and other parameters, which can well solve the problems of poor traditional drug loading, difficult manufacturing process, and early leakage or slow release of drugs. We injected SCH into the tumor tissue of mice, and under the illumination at 808nm, CuS NPs converted the near-infrared laser into heat to realize photothermal therapy, and at the same time, the agarose hydrogel was changed into a sol state and CPT was released of H_2_O_2_ inside the tumor, and realizes the self-supply of H_2_O_2_. At the same time, CuS NPs can accelerate the release of Cu^2+^ in an acidic environment and light, combined with H_2_O_2_ generated by CPT for chemokinetic treatment, and deplete glutathione inside the tumor. The SCH system we constructed achieved an extremely high tumor inhibition rate *in vitro* and *in vivo*, presenting a new idea for the design of future chemical kinetic systems.

## Results and discussion

**FIGURE 1:**
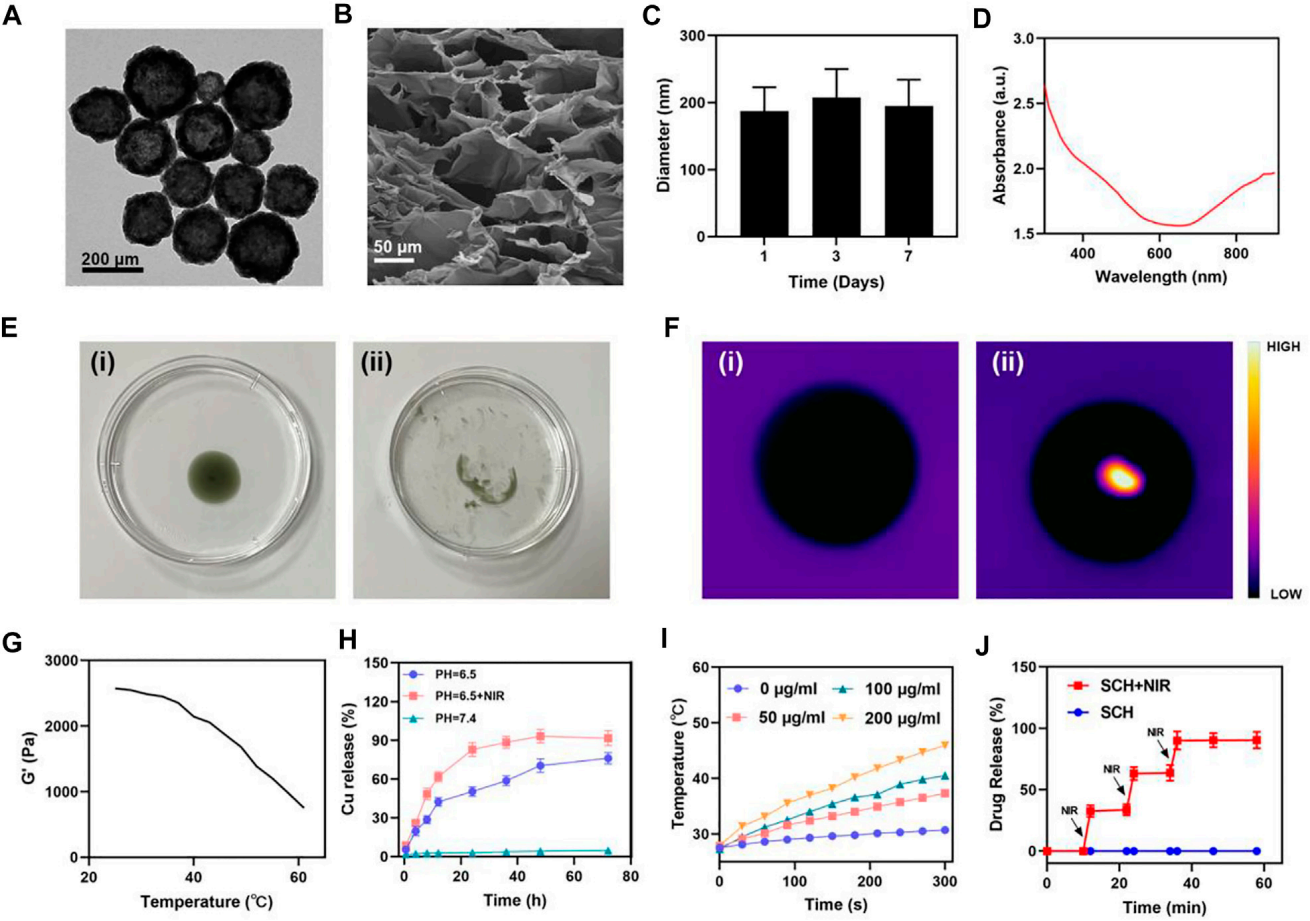
Characterization of SCH. **(A)** TEM image of CuS NPs. **(B)** SEM image of the SCH. **(C)** To determine the hydrodynamic diameter of CuS NPs (n = 3), DLS was employed. **(D)** CuS NPs absorbance spectra. **(E)**The morphology and **(F)**infrared thermal images of the prepared SCH before and after 0.5 W cm^−2^ 808 nm laser irradiation for 10 min **(G)**Temperature-dependent Rheological curves for the prepared SCH. **(H)**Time-dependent Cu^2+^ release from the SCH dispersed after different treatments. **(I)** Temperature changes of CuS NPs at various concentrations under a 5 min irradiation from an 808 nm laser at 0.5 W cm^−2^. **(J)** Black arrows are utilized to denote the irradiation time points (n = 3) in an *in vitro* SCH release profile with and without 808 nm laser irradiation.

A previously described simple two-step synthesis procedure was employed for the synthesis of the CuS NPs (Ding et al., 2022). CuS NPs with an average particle size of about 190 nm are shown in (transmission electron microscope (TEM) images in Figure 1A. The pharmacological impact of NPs is limited since kidneys can easily remove NPs that are less than 10 nm in size. Thus, the hydrogel drug delivery system can significantly improve the usefulness of CuS NPs. The average hydrodynamic size of CuS NPs as determined by dynamic light scattering (DLS) measurement is approximately 196 ± 39 nm (Figure 1C), which is in good agreement with the TEM findings. The FDA has approved agarose hydrogel as a safe substance that dissolves easily, has no hazardous effects on the body, and can be metabolized by the natural metabolic processes of the body. We prepared the SCH system by mixing low melting point agarose hydrogel with CuS NPs and CPT in certain proportions with constant stirring at 60°. Figure 1B displays the scanning electron microscopy (SEM) image of the as-prepared SCH. When maintained at room temperature after preparation, SCH is fairly stable. Because CuS NPs can facilitate the transformation of light energy to heat energy, a rise in temperature results. A wide absorption region in the near-infrared region is visible in the UV-Vis absorption spectrum, which confirms that CuS NPs have an absorption value at 808 nm (Figure 1D). Consequently, SCH will slowly release CuS NPs under continuous laser irradiation, causing the solution to turn turbid (Figure 1E). The temperature difference between the pre- and post-irradiation states was authenticated by employing infrared thermal imaging (Figure 1F). As the temperature rises, SCH will progressively soften, and its storage modulus will continue to decline, according to the rheological measurement results of SCH at various temperatures (Figure 1G). This outcome is in line with the hydrogel’s rheological characteristics. Additionally, we employed inductively coupled plasma-optical emission spectrometry (ICP-OES) to analyze how the laser affected the release of copper ions (Figure 1H), showing the NIR laser’s ability to accelerate copper ion release in an acidic environment. SCH was produced with CuS NPs in a variety of concentrations (0, 50, 100, and 200 μg/ml) to examine their photothermal performance. Assuming no other variables change, Figure 1I demonstrates that when the concentration of CuS NPs rises, the heating impact also increases. After 5 min of laser irradiation, the temperature of SCH with 200 mg/ml CuS NPs elevated by around 18.0°C. The findings of our subsequent test of SCH’s ability to control drug release are shown in Figure 1J. SCH can partly undergo disintegration under laser irradiation and releases the CPT. Following the cessation of laser irradiation, the hydrogel cools and solidifies, protecting the drug. Our SCH system’s potent capacity to control drug release is also shown by the fact that the drug is typically released entirely after four laser switching cycles.

**FIGURE 2:**
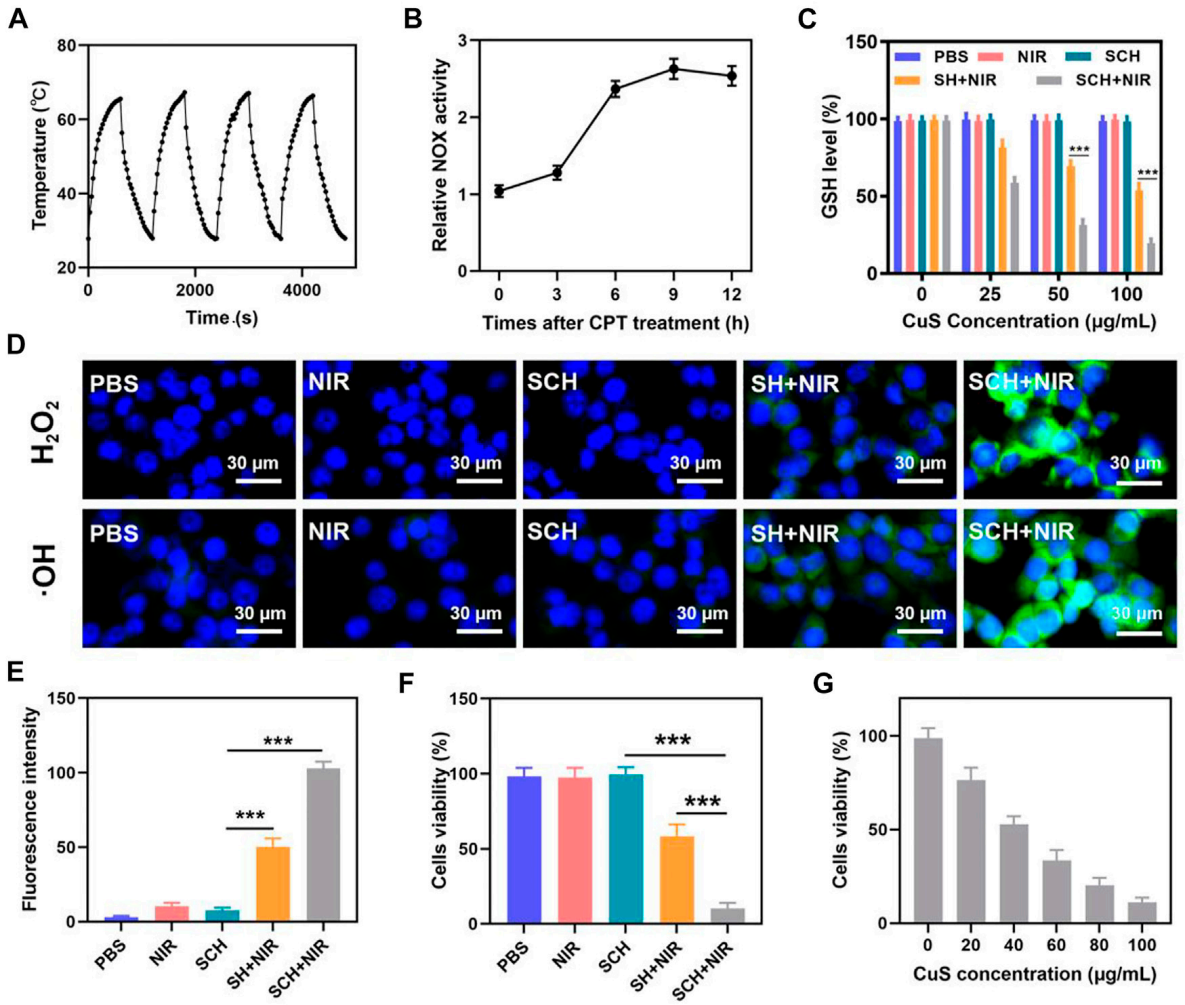
**(A)**Heating curve of SCH for four cycles having a 0.5 W cm^−2^ power intensity under irradiation by 808 nm laser. **(B)** NADPH oxidase (NOX) activity assessment in CT26 cells following 10 μg ml^−1^ CPT treatment at various time points. **(C)**The effect of different formulations on the intracellular GSH levels was determined by employing a GSH assay kit. (n = 3). **(D)** Confocal laser scanning microscopy images of different ROS produced in CT26 cells following various treatments. (n = 3). **(E)** Fluorescence intensity of OH from Figure 2D. (n = 3). **(F)** Various treatments’ *in vitro* cytotoxicity towards CT26 cells. (n = 3).**(G)** Cytotoxicity of various concentrations of CuS NPs on CT26 cells. (n = 3).*p < 0.05, **p < 0.01, ***p < 0.005; Student’s t-test.

Photothermal stability is a highly crucial aspect to consider when assessing photothermal agents (PTAs) (Liu et al., 2018; Ding et al., 2020; Chen et al., 2021d). Before verifying the tumor cell-killing effect of SCH system, we further evaluated the photothermal stability of SCH. An 808 nm NIR laser was utilized to constantly heat the SCH, and the switch was turned off after 10 min to let the SCH cool naturally to room temperature (Figure 2A). Four cycles of heating and cooling were carried out. The temperature graph demonstrates that the SCH peak temperature does not vary significantly, and the cooling trend is also consistent. In addition, we also calculated the photothermal conversion efficiency of CuS to be about 30.2% according to the previously reported method, which indicates that CuS has good photothermal performance. According to these results, the SCH has remarkable photothermal stability. After CPT treatment, a NOX activity detection kit was employed to confirm that the NOX in CT26 cells had been activated (Figure 2B). The anti-tumor activity based on ROS is inhibited by GSH’s reaction with reactive oxygen species to be oxidized, which produces GSSG (Franco et al., 2007; Zhu et al., 2022d). Due to glutathione deficiency, free radicals damage cells’ redox balance, induce oxidative stress and eventually result in cell apoptosis. We have established how SCH works in conjunction with NIR radiation for consuming GSH (Figure 2C). The findings demonstrate that Cu^2+^ may effectively lower intracellular GSH levels, while CPT can also generate a certain quantity of H_2_O_2_ to encourage GSH consumption, further enhancing the therapeutic effects of CDT. We utilized a hydrogen peroxide test kit as the probe for detecting intracellular H_2_O_2_ and hydroxyphenyl fluorescein (HPF) as the probe for detecting intracellular •OH to determine whether SCH may increase the generation of H_2_O_2_ in CT26 cells. The hydrogel coated with CuS (SH) was prepared to compare the experimental results. The control group’s and SCH group’s HPF fluorescence signals were both essentially nonexistent (Figures 2D,E). The SH in combination with the NIR group created a modest fluorescence effect, however, the SCH + NIR motivated the strongest green fluorescence because the SCH system yielded CPT under 808 nm irradiation, which then caused H_2_O_2_ to break down to produce •OH. We then administered various treatment combinations to CT26 tumor cells to verify the cytotoxicity of the SCH therapy. The viability of these cells was then evaluated via an MTT assay. In line with the inability of PBS, NIR, and SCH therapies in stopping the growth of the tumor, cells in these treatment groups did not demonstrate any discernible cytotoxicity (Figure 2F). On the other hand, SH + NIR and SCH + NIR treatments significantly increased the tumor cell cytotoxicity, with SCH + NIR treatment proving to be more cytotoxic than SH + NIR treatment alone. This is explained by the fact that CPT in the hydrogel system disintegrated H_2_O_2_ to yield •OH, which further enhanced the efficacy of CDT, thereby inhibiting tumor growth. As a result, this combination therapy strategy has the ability to compensate for the tumor’s deficiency of H_2_O_2_ and thereby improve treatment efficacy. As demonstrated in Figure 2G, we also developed SCH systems with various drug loading ratios and carried out MTT assays. The cell death was further boosted as CuS’s concentration elevated, showing that SCH’s cell killing was concentration-dependent.

**FIGURE 3:**
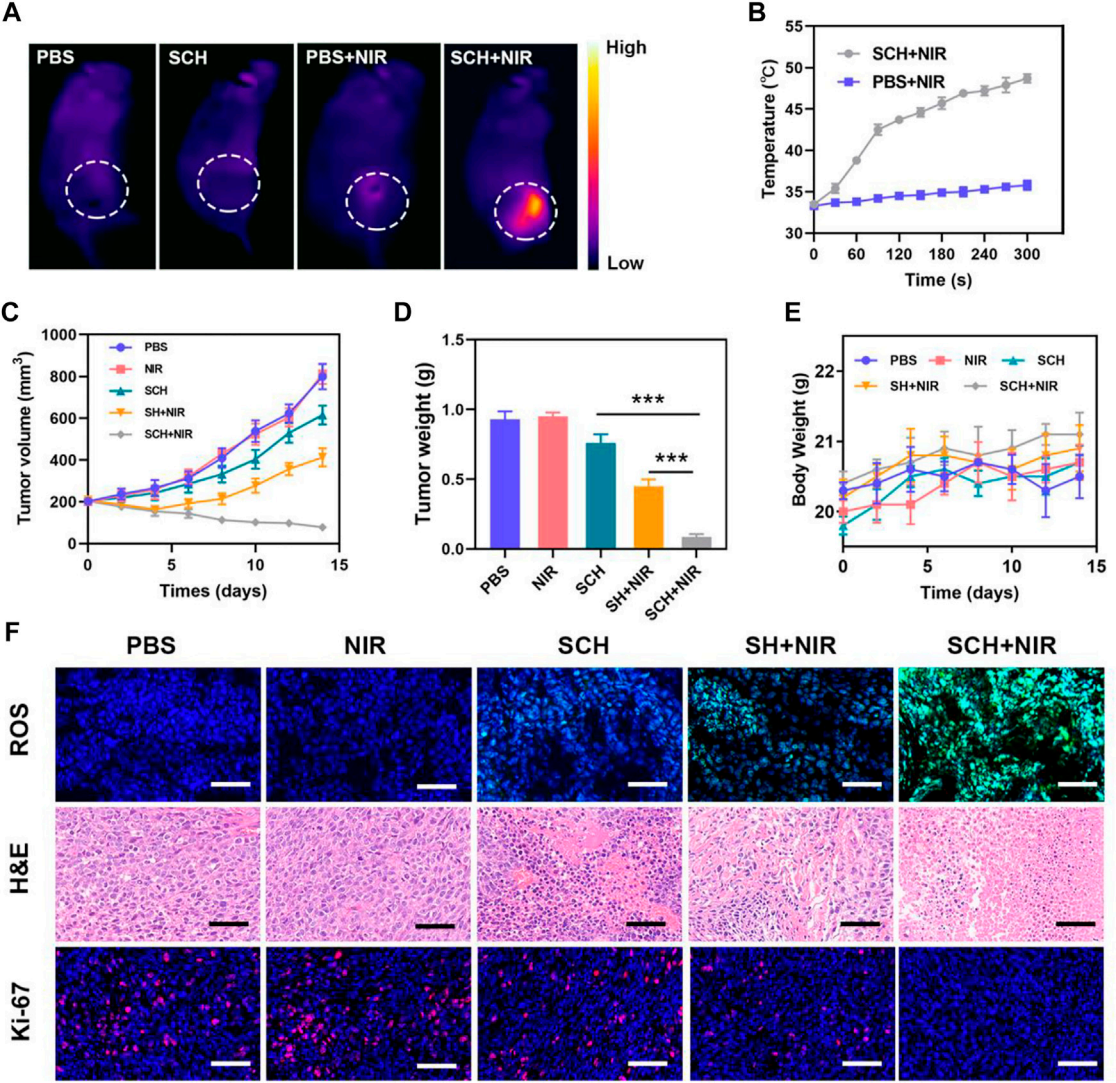
**(A)**Infrared thermal images of tumors in the specified treatment groups. **(B)** After being irradiated with 808 nm laser (0.5 W cm^−2^) for 5 min, the temperature elevates in mice having CT26 tumor, in the specified treatment groups. **(C)** The treatment groups’ tumor volume alters over time, as specified (n = 5). **(D)** Average tumor weight values associated with the indicated treatments (n = 5). **(E)** Changes in body weight in response to the indicated treatments (n = 5). **(F)** HPF, H&E, and Ki-67-stained tumor sections from the indicated treatment groups (n = 5). Scale bars: 100 μm ***p < 0.005; Student’s t-test. The results were presented as mean ± SD.

We then examined this prepared SCH’s capacity to induce the *in vivo* elimination of CT26 breast tumors in mice because they demonstrated positive antitumor activity *in vitro*. Following PBS or SCH treatment, the tumor site’s temperature was measured for 5 min under an 808 nm (0.5 W cm^−2^) laser. Temperatures in the SCH group were significantly higher than in the control group, according to IR thermal imaging (Figures 3A,B). The increase in temperature in the control group was insignificant (from 33.3 to 35.8°C), indicating that the increase in temperature in the SCH group was from 33.5 to 48.7°C. Since the heat resistance of tumor tissue is comparatively inferior to that of normal cells, SCH-mediated photothermal therapy can lead to the destruction of proteins and other active substances in tumor cells at elevated temperatures (42–47°C), thereby inducing apoptosis. On the other hand, SCH can release CuS NPs and CPT at high temperatures, enabling multiple rounds of treatments. The primary impact of SCH was determined by subcutaneously injecting 1 × 10^6^ CT26 cells into BALB/c mice. The mice received treatment after their main tumor volume had reached almost 200 mm^3^. Five groups of mice having tumors were created at random (5 mice in each group) (Zhang et al., 2020) PBS; (Chen et al., 2021a); NIR; (Zhu et al., 2021a); SCH; (Zhu et al., 2020); SH + NIR; and (Zhu et al., 2021b) SCH + NIR. The CuS NPs concentration was 20 mg/kg in groups 3, 4, and 5. After that, for 10 min, mice in groups 2, 4, and 5 were exposed to 808 nm laser radiation (0.5 W cm^−2^). Every 2 days, the weight of the mice was recorded. The tumor volumes in the control group (PBS) and the NIR group dramatically increased throughout the continuous treatment cycle, whereas the SCH group showed a slight tumor suppressor effect, because the hydrogel would degrade slowly in the animal body in the absence of light, releasing a small part of drugs to cause a certain degree of killing effect on the tumor. A better therapeutic impact is exhibited by the SH + NIR group. Though SH in combination with the laser has a certain tumor-ablating impact, the intracellular H_2_O_2_ level restricts SH’s ability to further induce the CDT effect. The group receiving treatment with SCH and NIR demonstrated the most effective tumor suppression (Figure 3C). Possibly following the laser irradiation, the chemotherapeutic drug CPT might boost H_2_O_2_ levels and further improve CuS NPs-mediated •OH generation in addition to promoting tumor cell apoptosis. Moreover, Cu^2+^ also could decrease intracellular GSH to cause oxidative stress damage. The weight of the tumor in mice following therapy matched the outcomes (Figure 3D). Throughout treatment, the mice’s body weight did not alter abnormally, proving that the agarose hydrogel was safe and nontoxic and that our course of treatment was safe (Figure 3E). Moreover, when the production of •OH in these mice was assessed using HPF, considerably elevated levels were determined in mice treated with SCH + NIR, suggesting that the current treatment approach can improve the CDT effectiveness and thus inhibit tumor growth. After the SCH + NIR group received therapy, Ki-67 staining was performed, and it was seen to be reduced. The hematoxylin and eosin (H&E) staining results showed that upon treatments with SCH + NIR, the solid tumor tissue’s structure was damaged, numerous tumor cells were necrotic, and the tissue’s cells were constricted with vanished nuclei (Figure 3F). These findings indicate that laser irradiation and our CFH could work together to treat tumors.

**FIGURE 4:**
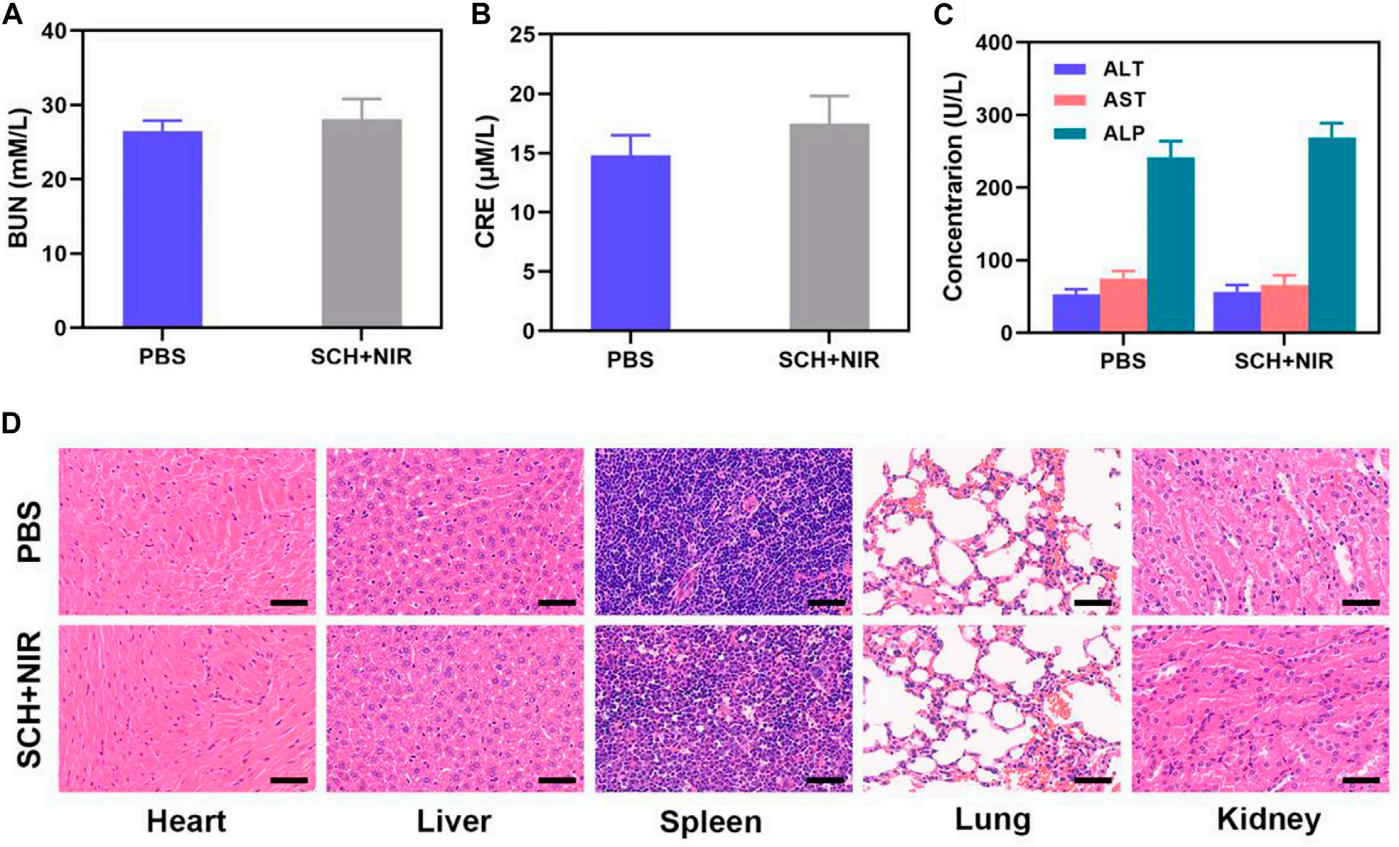
**(A)** Kidney function markers: BUN, **(B)** CRE, and **(C)** Liver function indicators: ALT, AST, and ALP following different treatments. (n = 3). **(D)** Results of histopathological examination (H&E-stained images) of the mice’s major organs, including the heart, lung, liver, kidneys, and spleen, after exposure to various therapies 14 days after the injection. (n = 3). Scale bars: 50 µm.

We then assessed liver and renal functions which were not impaired and this ensured that there was no systemic toxicity caused by SCH (Figures 4A–C). Also, the outcomes of comprehensive histological examinations of H&E- stained lung, liver, heart, spleen, and kidney sections from these animals confirmed that there are no major anomalies in any mice (Figure 4D). Therefore, in these mice, SCH therapy did not cause any severe adverse events. In summary, we have demonstrated that the SCH system can achieve extremely high tumor suppression rates. In the treatment of tumors, CDT has been limited by insufficient hydrogen peroxide content in tumors. Our SCH system provides new ideas for CDT treatment of tumors in the future.

## Conclusion

To conclude, we designed CuS NPs and CPT co-loaded thermosensitive injectable hydrogel with self-supplied H_2_O_2_ for enhanced Chemodynamic Therapy (SCH). We injected SCH into the tumor tissue of mice, and under the illumination at 808 nm, CuS NPs converted the near-infrared laser into heat to realize photothermal therapy, and at the same time, the agarose hydrogel was changed into a sol state and CPT was released. CPT activates nicotinamide adenine dinucleotide phosphate oxidase, increases the level of H_2_O_2_ inside the tumor, and realizes the self-supply of H_2_O_2_. At the same time, CuS NPs can accelerate the release of Cu^2+^ in an acidic environment and light, combined with H_2_O_2_ generated by CPT for chemokinetic treatment, and deplete glutathione inside the tumor. The SCH system we constructed achieved an extremely high tumor inhibition rate *in vitro* and *in vivo*, presenting a new idea for the design of future chemical kinetic systems. In the future, our system can be expected to be used in combination with advanced nanotechnology and immune technology concepts such as immunotherapy to further improve our therapeutic effect.

## Data Availability

The original contributions presented in the study are included in the article/Supplementary Material, further inquiries can be directed to the corresponding author.

## References

[B1] Chen H. Zheng D. Pan W. Li X. Lv B. Gu W. (2021). Biomimetic nanotheranostics camouflaged with cancer cell membranes integrating persistent oxygen supply and homotypic targeting for hypoxic tumor elimination. ACS Appl. Mat. Interfaces 13 (17), 19710–19725. 10.1021/acsami.1c03010 33890760

[B2] Chen L. Huang Q. Zhao T. Sui L. Wang S. Xiao Z. (2021). Nanotherapies for sepsis by regulating inflammatory signals and reactive oxygen and nitrogen species: New insight for treating COVID-19. Redox Biol. 45, 102046. 10.1016/j.redox.2021.102046 34174559 PMC8205260

[B3] Chen N. Fu W. Zhou J. Mei L. Yang J. Tian Y. (2021). Mn2+-doped ZrO2@PDA nanocomposite for multimodal imaging-guided chemo-photothermal combination therapy. Chin. Chem. Lett. 32 (8), 2405–2410. 10.1016/j.cclet.2021.02.030

[B4] Chen X. Chen Y. Wang C. Jiang Y. Chu X. Wu F. (2021). NIR-triggered intracellular H+ transients for lamellipodia-collapsed antimetastasis and enhanced chemodynamic therapy. Angew. Chem. Int. Ed. 60, 21905–21910. 10.1002/anie.202107588 34322970

[B5] Cheng J. Zhu Y. Xing X. Xiao J. Chen H. Zhang H. (2021). Manganese-deposited iron oxide promotes tumor-responsive ferroptosis that synergizes the apoptosis of cisplatin. Theranostics 11 (11), 5418–5429. 10.7150/thno.53346 33859755 PMC8039957

[B6] Deng H. Zhang J. Yang Y. Yang J. Wei Y. Ma S. (2022). Chemodynamic and photothermal combination therapy based on dual-modified metal-organic framework for inducing tumor ferroptosis/pyroptosis. ACS Appl. Mat. Interfaces 14, 24089–24101. 10.1021/acsami.2c00574 35588091

[B7] Ding D. Mei Z. Huang H. Feng W. Chen L. Chen Y. (2022). Oxygen-Independent sulfate radical for stimuli-responsive tumor nanotherapy. Adv. Sci. (Weinh). 9, e2200974. 10.1002/advs.202200974 35488513 PMC9189647

[B8] Ding K. Zheng C. Sun L. Liu X. Yin Y. Wang L. (2020). NIR light-induced tumor phototherapy using ICG delivery system based on platelet-membrane-camouflaged hollow bismuth selenide nanoparticles. Chin. Chem. Lett. 31 (5), 1168–1172. 10.1016/j.cclet.2019.10.040

[B9] Dong Z. Feng L. Chao Y. Hao Y. Chen M. Gong F. (2018). Amplification of tumor oxidative stresses with liposomal Fenton catalyst and glutathione inhibitor for enhanced cancer chemotherapy and radiotherapy. Nano Lett. 19, 805–815. 10.1021/acs.nanolett.8b03905 30592897

[B10] Feng L. Liu B. Xie R. Wang D. Qian C. Zhou W. (2020). An ultrasmall SnFe2O4 nanozyme with endogenous oxygen generation and glutathione depletion for synergistic cancer therapy. Adv. Funct. Mat. 31 (5), 2006216. 10.1002/adfm.202006216

[B11] Fenton H. J. H. (1894). LXXIII.—oxidation of tartaric acid in presence of iron. J. Chem. Soc. Trans. 65 (0), 899–910. 10.1039/CT8946500899

[B12] Franco R. Panayiotidis M. I. Cidlowski J. A. (2007). Glutathione depletion is necessary for apoptosis in lymphoid cells independent of reactive oxygen species formation. J. Biol. Chem. 282 (42), 30452–30465. 10.1074/jbc.M703091200 17724027 PMC2267748

[B13] Fu J. Li T. Yang Y. Jiang L. Wang W. Fu L. (2021). Activatable nanomedicine for overcoming hypoxia-induced resistance to chemotherapy and inhibiting tumor growth by inducing collaborative apoptosis and ferroptosis in solid tumors. Biomaterials 268, 120537. 10.1016/j.biomaterials.2020.120537 33260096

[B14] Li X. Hetjens L. Wolter N. Li H. Shi X. Pich A. (2022). Charge-reversible and biodegradable chitosan-based microgels for lysozyme-triggered release of vancomycin. J. Adv. Res. 1, 1. 10.1016/j.jare.2022.02.014 PMC9811367 36585117

[B15] Li X. Kong L. Hu W. Zhang C. Pich A. Shi X. (2022). Safe and efficient 2D molybdenum disulfide platform for cooperative imaging-guided photothermal-selective chemotherapy: A preclinical study. J. Adv. Res. 37, 255–266. 10.1016/j.jare.2021.08.004 35499043 PMC9039738

[B16] Li X. Li H. Zhang C. Pich A. Xing L. Shi X. (2021). Intelligent nanogels with self-adaptive responsiveness for improved tumor drug delivery and augmented chemotherapy. Bioact. Mat. 6 (10), 3473–3484. 10.1016/j.bioactmat.2021.03.021 PMC8024537 33869898

[B17] Li X. Luo R. Liang X. Wu Q. Gong C. (2021). Recent advances in enhancing reactive oxygen species based chemodynamic therapy. Chin. Chem. Lett. 33, 2213–2230. 10.1016/j.cclet.2021.11.048

[B18] Li X. Ouyang Z. Li H. Hu C. Saha P. Xing L. (2021). Dendrimer-decorated nanogels: Efficient nanocarriers for biodistribution *in vivo* and chemotherapy of ovarian carcinoma. Bioact. Mat. 6 (10), 3244–3253. 10.1016/j.bioactmat.2021.02.031 PMC7970313 33778202

[B19] Li X. Xing L. Hu Y. Xiong Z. Wang R. Xu X. (2017). An RGD-modified hollow silica@Au core/shell nanoplatform for tumor combination therapy. Acta Biomater. 62, 273–283. 10.1016/j.actbio.2017.08.024 28823719

[B20] Li X. Xing L. Zheng K. Wei P. Du L. Shen M. (2017). formation of gold nanostar-coated hollow mesoporous silica for tumor multimodality imaging and photothermal therapy. ACS Appl. Mat. Interfaces 9 (7), 5817–5827. 10.1021/acsami.6b15185 28118704

[B21] Li Y. Lu W. Huang Q. Li C. Chen W. (2010). Copper sulfide nanoparticles for photothermal ablation of tumor cells. Nanomedicine 5 (8), 1161–1171. 10.2217/nnm.10.85 21039194

[B22] Lin L. Wang S. Deng H. Yang W. Rao L. Tian R. (2020). Endogenous labile iron pool-mediated free radical generation for cancer chemodynamic therapy. J. Am. Chem. Soc. 142 (36), 15320–15330. 10.1021/jacs.0c05604 32820914

[B23] Liu W. Xiang H. Tan M. Chen Q. Jiang Q. Yang L. (2021). Nanomedicine enables drug-potency activation with tumor sensitivity and Hyperthermia Synergy in the Second near-infrared Biowindow. ACS Nano 15 (4), 6457–6470. 10.1021/acsnano.0c08848 33750100

[B24] Liu X. Ren Q. Fu F. Zou R. Wang Q. Xin G. (2015). CuS@mSiO2-PEG core-shell nanoparticles as a NIR light responsive drug delivery nanoplatform for efficient chemo-photothermal therapy. Dalton Trans. 44 (22), 10343–10351. 10.1039/c5dt00198f 25970690

[B25] Liu Y. Zhai S. Jiang X. Liu Y. Wang K. Wang C. (2021). Intracellular mutual promotion of redox homeostasis regulation and iron metabolism disruption for enduring chemodynamic therapy. Adv. Funct. Mat. 31 (17), 2010390. 10.1002/adfm.202010390

[B26] Liu Y. Zhen W. Jin L. Zhang S. Sun G. Zhang T. (2018). All-in-One theranostic nanoagent with enhanced reactive oxygen species generation and modulating tumor microenvironment ability for effective tumor eradication. ACS Nano 12 (5), 4886–4893. 10.1021/acsnano.8b01893 29727164

[B27] Meng X. Zhou K. Qian Y. Liu H. Wang X. Lin Y. (2022). Hollow cuprous Oxide@Nitrogen-doped carbon nanocapsules for cascade chemodynamic therapy. Small 18, e2107422. 10.1002/smll.202107422 35233936

[B28] Sang Y. Cao F. Li W. Zhang L. You Y. Deng Q. (2020). Bioinspired construction of a nanozyme-based H2O2 homeostasis disruptor for intensive chemodynamic therapy. J. Am. Chem. Soc. 142 (11), 5177–5183. 10.1021/jacs.9b12873 32100536

[B29] Tang Y. Lu X. Yin C. Zhao H. Hu W. Hu X. (2019). Chemiluminescence-initiated and *in situ*-enhanced photoisomerization for tissue-depth-independent photo-controlled drug release. Chem. Sci. 10 (5), 1401–1409. 10.1039/c8sc04012e 30809357 PMC6354828

[B30] Wang J. Sui L. Huang J. Miao L. Nie Y. Wang K. (2021). MoS2-based nanocomposites for cancer diagnosis and therapy. Bioact. Mat. 6 (11), 4209–4242. 10.1016/j.bioactmat.2021.04.021 PMC8102209 33997503

[B31] Wang Q. Gao Z. Zhao K. Zhang P. Zhong Q.-Z. Yu Q. (2021). Co-delivery of enzymes and photosensitizers via metal-phenolic network capsules for enhanced photodynamic therapy. Chin. Chem. Lett. 33, 1917–1922. 10.1016/j.cclet.2021.11.040

[B32] Wei Q. Chen Y. Ma X. Ji J. Qiao Y. Zhou B. (2018). High-Efficient clearable nanoparticles for multi-modal imaging and image-guided cancer therapy. Adv. Funct. Mat. 28 (2), 1704634. 10.1002/adfm.201704634

[B33] Yan J. Zhang Y. Zheng L. Wu Y. Wang T. Jiang T. (2022). Let-7i miRNA and platinum loaded nano-graphene oxide platform for detection/reversion of drug resistance and synergetic chemical-photothermal inhibition of cancer cell. Chin. Chem. Lett. 33 (2), 767–772. 10.1016/j.cclet.2021.08.018

[B34] Zhang K. Yu Z. Meng X. Zhao W. Shi Z. Yang Z. (2019). A bacteriochlorin-based metal–organic framework nanosheet superoxide radical generator for photoacoustic imaging-guided highly efficient photodynamic therapy. Adv. Sci. 6 (14), 1900530. 10.1002/advs.201900530 PMC6661935 31380214

[B35] Zhang X. Ong'achwa Machuki J. Pan W. Cai W. Xi Z. Shen F. (2020). Carbon nitride hollow theranostic nanoregulators executing laser-activatable water splitting for enhanced ultrasound/fluorescence imaging and cooperative phototherapy. ACS Nano 14 (4), 4045–4060. 10.1021/acsnano.9b08737 32255341

[B36] Zhao T. Wu W. Sui L. Huang Q. Nan Y. Liu J. (2022). Reactive oxygen species-based nanomaterials for the treatment of myocardial ischemia reperfusion injuries. Bioact. Mat. 7, 47–72. 10.1016/j.bioactmat.2021.06.006 PMC8377441 34466716

[B37] Zhou Q. M. Lu Y. F. Zhou J. P. Yang X. Y. Wang X. J. Yu J. N. (2021). Self-amplification of oxidative stress with tumour microenvironment-activatable iron-doped nanoplatform for targeting hepatocellular carcinoma synergistic cascade therapy and diagnosis. J. Nanobiotechnology 19 (1), 361. 10.1186/s12951-021-01102-0 34749740 PMC8576982

[B38] Zhu D. Chen H. Huang C. Li G. Wang X. Jiang W. (2022). H_2_O_2_ self-producing single-atom nanozyme hydrogels as light-controlled oxidative stress amplifier for enhanced synergistic therapy by transforming “cold” tumors. Adv. Funct. Mat. 32, 2110268. 10.1002/adfm.202110268

[B39] Zhu D. Duo Y. Meng S. Zhao Y. Xia L. Zheng Z. (2020). Tumor-exocytosed exosome/aggregation-induced emission luminogen hybrid nanovesicles facilitate efficient tumor penetration and photodynamic therapy. Angew. Chem. Int. Ed. 59, 13836–13843. 10.1002/anie.202003672 32367646

[B40] Zhu D. Ling R. Chen H. Lyu M. Qian H. Wu K. (2022). Biomimetic copper single-atom nanozyme system for self-enhanced nanocatalytic tumor therapy. Nano Res. 15, 7320–7328. 10.1007/s12274-022-4359-6

[B41] Zhu D. Zhang J. Luo G. Duo Y. Tang B. Z. (2021). Bright bacterium for hypoxia-tolerant photodynamic therapy against orthotopic colon tumors by an interventional method. Adv. Sci. (Weinh). 8, 2004769. 10.1002/advs.202004769 PMC8336512 34145986

[B42] Zhu D. Zhang T. Li Y. Huang C. Suo M. Xia L. (2022). Tumor-derived exosomes co-delivering aggregation-induced emission luminogens and proton pump inhibitors for tumor glutamine starvation therapy and enhanced type-I photodynamic therapy. Biomaterials 283, 121462. 10.1016/j.biomaterials.2022.121462 35272223

[B43] Zhu D. Zheng Z. Luo G. Suo M. Li X. Duo Y. (2021). Single injection and multiple treatments: An injectable nanozyme hydrogel as AIEgen reservoir and release controller for efficient tumor therapy. Nano Today 37, 101091. 10.1016/j.nantod.2021.101091

[B44] Zhu Y. Zhao T. Liu M. Wang S. Liu S. Yang Y. (2022). Rheumatoid arthritis microenvironment insights into treatment effect of nanomaterials. Nano Today 42, 101358. 10.1016/j.nantod.2021.101358

