# Supplementary material for: CuS nanoparticles and camptothecin co-loaded thermosensitive injectable hydrogel with self-supplied H2O2 for enhanced chemodynamic therapy
Source: Front Bioeng Biotechnol. 2022 Aug 29;10:1003777. doi: 10.3389/fbioe.2022.1003777 (PMC9465046; doi:10.3389/fbioe.2022.1003777)
Supplement: Supplementary file 1 [file DataSheet1.docx]

Experimental Procedures

**Materials and reagents.**

Copper (II) chloride dihydrate (CuCl2·2H2O) and hydrazine hydrate aqueous solution (N2H4·H2O) were purchased from Sinopharm Chemical Reagent Co., Ltd. (China). Sodium sulfide nonahydrate (Na2S·9H2O), poly(vinylpyrrolidone) (PVP K40, Mw = 40,000), 3’-(4-hydroxyphenyl) fluorescein (HPF) and Hydrogen peroxide (H_2_O_2_) fluorescence detection kit were purchased from Sigma-Aldrich. Agarose was purchased from Yare Shanghai. The reduced GSH assay kit was purchased from Nanjing Jiancheng Bioengineering Institute. The other reagents used in this work were purchased from Sinopharm Chemical Reagent (China) and Aladdin-Reagent (China).

**Cell culture**

CT26 mouse cancer cell line was obtained from the Cell Bank of the Chinese Academy of Sciences and incubated in RPMI-1640 medium supplemented with 10% FBS in a humidified atmosphere at 37℃.

**Preparation and characterization of Copper Sulfide Nanoparticles (CuS)[**[**1**](#_ENREF_1)**]**

Briefly, a CuCl_2_ solution (100 μL) and poly(vinylpyrrolidone) (0.24 g) were mixed in deionized water (25 mL) and magnetically stirred at room temperature (rt). Next, NaOH (pH 9, 25 mL) solution was transferred to the mixture, followed by the addition of hydrazine solution (6.4 μL), which resulted in the formation of a suspension of Cu_2_O spheres. Subsequently, a Na_2_S (320 mg mL−1, 200 μL) aqueous solution was added to the Cu_2_O suspension, stirred at 60 °C for 2 h before cooling to rt, and centrifuged (11 000 rpm, 10 min), followed by washing with deionized (DI) water (twice) to obtain CuS. The morphology structures of CuS were detected by the TEM (JEOL-2100). UV-vis spectra of samples were recorded by the UV-vis spectrophotometry Lambda 35 (Perkin-Elmer). Dynamic light scattering (DLS) and zeta potential measurements were conducted on a Zeta sizer Nano series (Nano ZS90, Malvern Instrument Ltd.).

**Preparation and characterization of SCH**

The general protocol for the hydrogel preparation is as follows. The prepared CuS (1.5 mg/mL in PBS) and CPT (0.25 mg/mL in a mixed solvent of DMSO and water (8: 2)) were added into 2% agarose solution to form SCH. Scanning electron microscopy (SEM) images were captured on a Hitachi FE-SEM S4800 instrument with an acceleration voltage of 3 kV. UV-vis spectra of samples were recorded by the UV-vis spectrophotometry Lambda 35 (Perkin-Elmer).

**Rheological Test**

Rheology experiments were performed on an Anton Paar rheometer. Hydrogel samples of different temperatures were prepared and gently placed on the middle of a 15 mm diameter parallel plate with a proper gap. Dynamic oscillatory frequency sweep measurements were conducted at a 1% strain amplitude. To prevent the evaporation of water, a lid was prepared on the top.

**Photothermal Conversion Ability**

An 808 nm NIR laser (Changchun New Industries Tech.Co., Ltd., Changchun, China) with irradiation powers was used to stimulate the concentrations of CuS (0, 50, 100, 200 ug/mL) in an aqueous medium. The photothermal images of the CuS-based suspensions during laser irradiation were recorded every 30 s using an infrared thermal imaging system. Heating curve of SCH for four cycles at a power intensity of 0.5 W cm^-2^ under 808nm laser was measured by the infrared thermal imaging system.

**CPT and Cu^2+^ release study**

The in vitro CPT release profile from SCH was carried out. 1mL of SCH containing 20 μg CPT was added into culture dish. To investigate the stimuli effect of laser irradiation on the release behavior, the release experiment of CPT was initially performed with or without 0.5 W/cm^2^ 808 nm laser irradiation for 3 min. At appropriate time point, 100 μL of different samples were collected, and an UV-vis spectrophotometer was used to monitor the released CPT content.

1mL of SCH containing 200 μg CPT was added into culture dish. To investigate the stimuli effect of laser irradiation and pH on the release behavior, the release experiment of CPT was initially performed with or without 0.5 W/cm^2^ 808 nm laser irradiation under different pH value for 10 min. At appropriate time point, 100 μL of different samples were collected for ICP-AES measurement.

**NADPH oxidase activity**

The activity of NADPH oxidase was determined in membrane fractions (50 μg of protein) incubated with 1 mM EGTA and 5 μM lucigenin in phosphate buffer, pH 7.0. The assay was initiated by the addition of 50 μM NADPH to the incubation mixture. Samples were counted immediately using a tabletop luminometer with sampling time every 6 s. Samples were counted over a period of 5 min, and the fluorescence values were recorded for over 2 min of stable readings and averaged for that sample.

***In vitro* anti-cancer effect of SCH**

Typically, CT26 cells were incubated in six-well plates at 37 °C with 5% CO_2_ for 24 h; afterward, the culture medium was replaced by new culture medium and the Afterwards, cells were incubated with 5 different groups at different CuS concentration: (1) PBS; (2) NIR; (3) SCH; (4) SH + NIR; (5) SCH + NIR. Then, cells in group 2, 4 and 5 were exposed to 808 nm laser radiation (0.5 W/cm^2^) for 5 min. After incubation for another 6 h. Finally, the viability of CT26 cells was determined by a CCK-8 cell cytotoxicity assay. The cell viability was normalized by control group without any treatment.

In addition, U937 and L02 cells were incubated in six-well plates at 37 °C with 5% CO2 for 24 h; afterward, the culture medium was replaced by new culture medium and the afterwards, cells were incubated with SCH at different CuS concentration. After incubation for another 6 h. Finally, the viability of U937 and L02 cells was determined by a CCK-8 cell cytotoxicity assay.

***In vitro* ROS generation**

ROS generation was also assessed in vitro on CT26 cells. The intracellular generation

of ROS was detected utilizing HPF and H_2_O_2_ detection kit. Afterwards, cells were incubated for 5 different groups: (1) PBS; (2) NIR; (3) SCH; (4) SH + NIR; (5) SCH + NIR. The CPT concentration was 20 μg/mL in group 3 and 5. Then, HPF or H_2_O_2_ detection kit were added. Then, cells in group 2, 4 and 5 were exposed to 808 nm laser radiation (0.5 W/cm^2^) for 5 min and detected under a fluorescent microscope (IX81, Olympus, Japan). Fluorescence intensity was measured by ImageJ software.

**Detection of Intracellular GSH.**

The commercially available GSH assay kit was used to detect the depletion of GSH. CT26 cells were incubated with 5 different group at different CuS concentration: (1) PBS; (2) NIR; (3) SCH; (4) SH + NIR; (5) SCH + NIR. Then, cells in group 2, 4 and 5 were exposed to 808 nm laser radiation (0.5 W/cm^2^) for 5 min. After 12 hours of incubation, the GSH content was measured by employing a commercial colorimetric GSH assay kit. The assay was carried out according to the manufacturer’s instructions. The absorbance of 340 nm was measured by a microplate reader.

**Animal tumor models**

Female BALB/c nude mice aged 4-5 week were purchased from Vital River Company (Beijing, China). 100 μL of CT26 cell suspension (1×10^6^ cells per mL) were subcutaneous injected into each mouse to establish the tumor models. The animal experiments were carried out according to the protocol approved by the Ministry of Health in People’s Republic of PR China and were approved by the Administrative Committee on Animal Research of the Wuhan University.

***In vivo* infrared thermography**

To monitor the in vivo photothermal effect, SCH was intratumorally injected into the tumor-bearing mice, and then the tumors suffered from 0.5 W/cm^2^ irradiation for 10 min at 1 h post-injection. PBS injection used as control group. Meanwhile, the temperature at the tumor was monitored using an infrared camera (Fotric 225).

***In vivo* antitumor study**

The mice were firstly divided randomly into 5 groups (each group included 5 mice): (1) PBS; (2) NIR; (3) SCH; (4) SH + NIR; (5) SCH + NIR. Then, cells in group 2, 4 and 5 were exposed to 808 nm laser radiation (0.5 W/cm2) for 5 min. The CuS dose was 20 mg/kg in group 3, 4, and 5. Then, cells in group 2, 4 and 5 were exposed to 808 nm laser radiation (0.5 W/cm^2^) for 10 min. The injection method is intratumoral injection. NIR was conducted 1h after the injection. Mice body weight and tumor volume in all groups were monitored every 2 days. A caliper was employed to measure the tumor length and tumor width and the tumor volume was calculated according to following formula. Tumor volume = tumor length × tumor width^2^ / 2. After 15 days treatment, mice were sacrificed. Five main organs (heart, liver, spleen, lung and kidney) of all mice were harvested, washed with PBS, and fixed with paraformaldehyde for histology analysis. The blood samples from these mice (≈1 mL) were collected for blood biochemistry analysis. And the tumor tissues were weighed, and fixed in 4% neutral buffered formalin, processed routinely into paraffin, and sectioned at 4 μm. Then the sections were stained with HPF, Ki-7 and HE and finally examined by using optical microscope (BX51, Olympus, Japan) and fluorescence microscope (IX81, Olympus, Japan).

**Statistical analysis**

Data analyses were conducted using the GraphPad Prism 5.0 software. Significance between every two groups was calculated by the student’s t-test. *P < 0.05, **P < 0.01, ***P < 0.005.

Supplementary figures


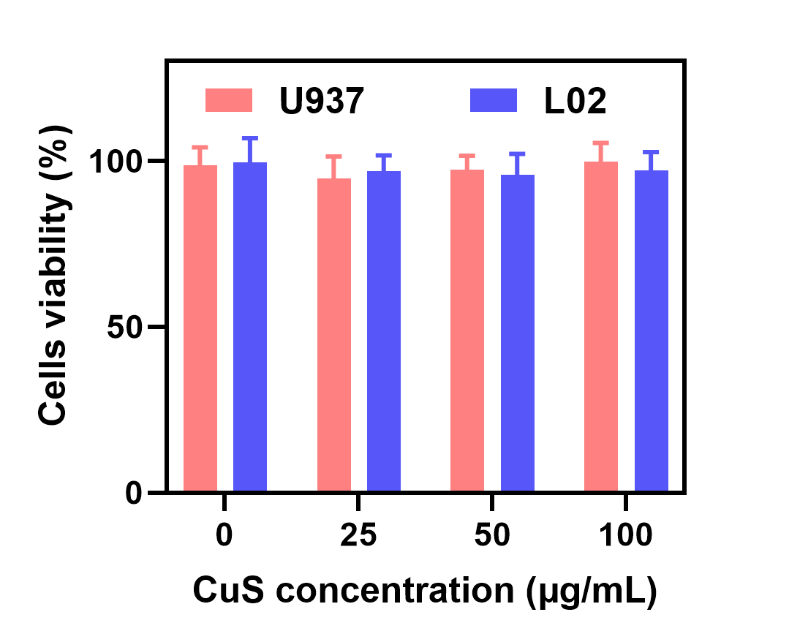


**Figure S1.** Cell viability of normal cells after SCH treatments.


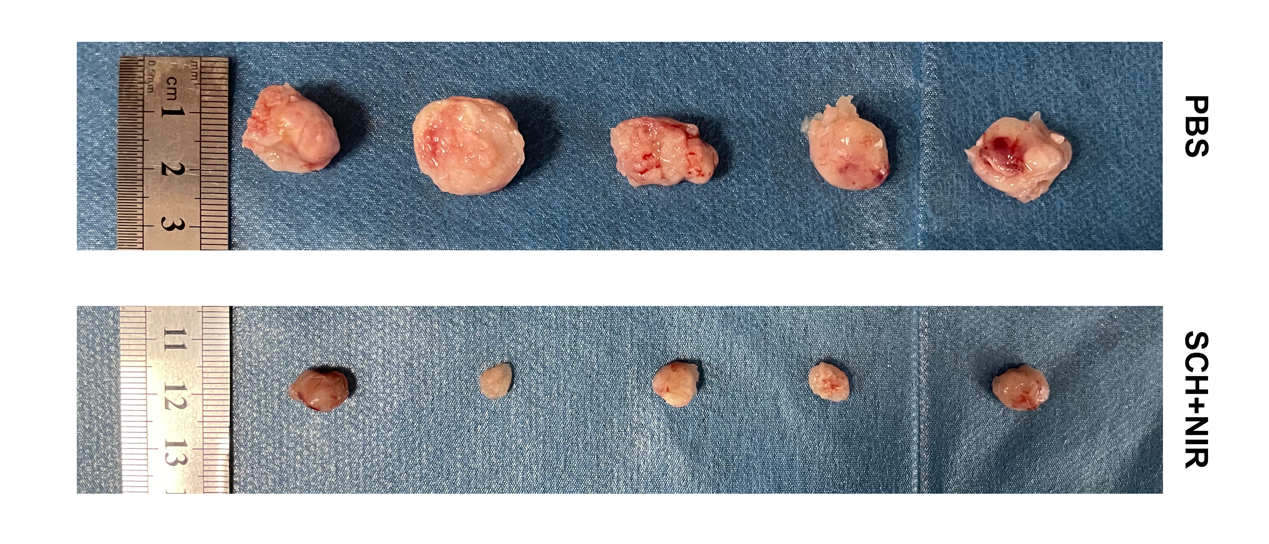


**Figure S2.** Photographs of tumors isolated from different groups after treatments.

**References**

[1] Liu W, Xiang H, Tan M, Chen Q, Jiang Q, Yang L, et al. Nanomedicine Enables Drug-Potency Activation with Tumor Sensitivity and Hyperthermia Synergy in the Second Near-Infrared Biowindow. ACS nano. 2021;15:6457-70.
